# Sex hormone modulation of cell growth and apoptosis of the human monocytic/macrophage cell line

**DOI:** 10.1186/ar1791

**Published:** 2005-07-21

**Authors:** Maurizio Cutolo, Silvia Capellino, Paola Montagna, Paola Ghiorzo, Alberto Sulli, Barbara Villaggio

**Affiliations:** 1 Research Laboratory and Division of Rheumatology, Department of Internal Medicine, University of Genova, Italy

## Abstract

Sex hormones seem to modulate the immune/inflammatory responses by different mechanisms in female and male rheumatoid arthritis patients. The effects of 17β-oestradiol and of testosterone were tested on the cultured human monocytic/macrophage cell line (THP-1) activated with IFN-γ in order to investigate their role in cell proliferation and apoptosis. Activated human THP-1 cells were cultured in the presence of 17β-oestradiol and testosterone (final concentration, 10 nM). The evaluation of markers of cell proliferation included the NF-κB DNA-binding assay, the NF-κB inhibition complex, the proliferating cell nuclear antigen expression and the methyl-tetrazolium salt test. Apoptosis was detected by the annexin V-propidium assay and by the cleaved poly-ADP ribose polymerase expression. Specific methods included flow analysis cytometry scatter analysis, immunocytochemistry and western blot analysis. Cell growth inhibition and increased apoptosis were observed in testosterone-treated THP-1 cells. Increased poly-ADP ribose polymerase-cleaved expression and decreased proliferating cell nuclear antigen expression, as well as an increase of IκB-α and a decrease of the IκB-α phosphorylated form (ser 32), were found in testosterone-treated THP-1 cells. However, the NF-κB DNA binding was found increased in 17β-oestradiol-treated THP-1 cells. The treatment with staurosporine (enhancer of apoptosis) induced decreased NF-κB DNA binding in all conditions, but particularly in testosterone-treated THP-1 cells. Treatment of THP-1 by sex hormones was found to influence cell proliferation and apoptosis. Androgens were found to increase the apoptosis, and oestrogens showed a protective trend on cell death – both acting as modulators of the NF-κB complex.

## Introduction

Experimental and clinical evidence indicates that immune reactivity is greater in females than in males and suggests that gonadal steroids may play an important role in the regulation of the immune response [1-4]. Indeed, many cells of the immune system have been found to possess functional sex hormone receptors, such as CD8-positive T cells, B cells and, notably, monocytes/macrophages [5,6].

Therefore, 17β-oestradiol (E2) was found to inhibit cellular apoptosis, to increase antibody production by B cells and to exert dose-related effects on T-cell functions [7]. Androgens seem to exert effects opposite to those of E2 on immune response [8]. Clinical epidemiology clearly confirms a higher prevalence of autoimmune diseases in female subjects when compared with male subjects [9].

The studies concerning the functional interaction between the NF-κB pathway and members of the steroid hormone receptor family, and their role in synovial inflammation, have advanced significantly, although with controversial results [10,11]. In particular, after binding with E2, oestrogen receptors have been shown to interact with NF-κB factors, via transcriptional co-factors, resulting in mutual or non-mutual antagonism. Other studies hypothesize that, since oestrogen receptors may repress both constitutive and inducible NF-κB activity, the overexpression of NF-κB-inducible genes in oestrogen receptor-negative cells might contribute to malignant cell growth and chemotherapeutic resistance [12,13]. On the contrary, further studies report that E2 blocks the transcriptional activity of p65 in macrophages [14]. However, these opposite observations arise using different cell lines (human/animals) and culture conditions as well as different hormone concentrations [15]. In addition, multiple mechanisms concerning the interaction between oestrogen receptors and NF-κB have been proposed, such as repression of NF-κB DNA binding by physical association with oestrogen receptors and the regulation of IκB-α expression by oestrogens [16,17].

The androgen receptor seems to be closely related to the glucocorticoid receptor in terms of both structure and sequence homology. The androgen receptor and the glucocorticoid receptor have been shown to interact and repress activator protein 1 via a similar mechanism; consequently, it would not be surprising that the androgen receptor might also interact with NF-κB in a manner very similar to that observed for the glucocorticoid receptor [18]. Both the androgen receptor and NF-κB are inducible transcription factors with some opposing functions in the regulation of immune and inflammatory responses [19].

It is possible that inflammatory agents that activate NF-κB *in vivo *may interfere with normal androgen signalling, and recent studies indicate that the androgen receptor and NF-κB (subunit p65) are mutual transcriptional antagonists [20]. The present study was therefore undertaken to examine the long-term (time course of 7 days) effects of sex hormones on activated cultured human monocytic/macrophage cell line (THP-1) cells by investigating their effects on cell proliferation and apoptosis. These cells are equipped with functional sex hormone receptors and are an important target of sex steroid hormones, particularly in inflammatory diseases such as rheumatoid arthritis (RA) [21]. In particular, in the present study we used pharmacological concentrations of E2 (final concentration, 10 nM; Sigma-Aldrich, Milan, Italy) that have been already described as the most efficient in stimulating macrophages *in vitro *[22]. Accordingly, the same concentration has been used for testosterone [23].

We therefore investigated sex hormone effects on the NF-κB pathway, as a complex of molecules modulating cellular responses in activated cells.

## Materials and methods

### Cell cultures and treatments

THP-1 cells (Interlab cell line collection HTL097014; IST c/o CBA, Genoa, Italy) were cultured in RPMI-1640 medium supplemented with 2% foetal bovine serum (Sigma-Aldrich) (5% CO_2 _humidified atmosphere at 37°C). Moreover, the absence of binding of the hormones with other foetal bovine serum components related to growth rate and the survival of cultured THP-1 cells over the course of 7 days were investigated. The cells were maintained in logarithmic growth by passage every 3–4 days. The viability of the cells (97–98%) was tested by the Trypan blue exclusion procedure. Briefly, the cells were seeded into six-well flat-bottom plates (10^6 ^cells/well) and were treated with 500 U/ml IFN-γ (Sigma-Aldrich) in order to differentiate THP-1 into macrophage cells [24]. The activation and transformation of the cells was evaluated by the expression of different macrophage antigens: CD68, CD14, HAM 56, Mac 387. The THP-1 activated cells were then incubated for 24, 48, 72, 96 and 168 hours with E2 and testosterone (10 nM).

After that time samples of THP-1 cells were also treated with an apoptosis inducer [25], staurosporine (17 nM; Sigma Aldrich), for 24 hours. At the end of the different incubation times, the cells were harvested, washed in Dulbecco's phosphate-buffered saline (DPBS) and treated with different lysis buffers for the nuclear and total protein extraction. Part of the cell was then collected for DNA content (normal and apoptotic) evaluation by flow analysis cytometry scatter analysis and for immunocytochemistry analysis.

### Expression of macrophage markers

The immunocytochemistry analysis to evaluate the expression of CD68, CD14, HAM56 and Mac387 in the treated cells showed positive results (data not shown), confirming the activation of monocytes by IFN-γ.

### Cell growth

The cell growth was evaluated at different times by the methyl-tetrazolium salt test, which represents a quantitative colorimetric assay to detect cell survival and proliferation. The test is based on the ability of living cells to cleave the tetrazolium ring at the level of active mitochondria. Briefly, the cells were seeded into 96-well microtitre plates and were treated according to the experimental design. At the established time, 50 μl methyl-tetrazolium salt labelling reagent [3-(4,5-dimethylthiazol-2-yl)-2,5-diphenyltetrazolium bromide, 5 mg/ml in PBS] was added to each well and incubated in humidified atmosphere at 37°C. Four hours later, 100 μl dimethylsulfoxide were introduced into each well and mixed thoroughly. The absorbance was calculated at 540 nm, using a scanning multiwell spectrophotometer. Standard curves were constructed for THP-1 cells using known plating densities, allowing the cell number to be calculated from this optical density reading. Each experiment was performed in triplicate.

### Apoptosis

The apoptotic events were evaluated after 168 hours from both sex hormone stimulation and staurosporine treatment (17 nM for 24 hours) by annexin V-propidium iodide analysis (MBL Co., Ltd, Nagoya, Japan), in order to detect the early-stage apoptosis (cells only annexin-positive) and the late-stage apoptosis (cells annexin-positive and propidium-positive). After resuspending the cells (1 × 10^5^) into 200 μl of 1 x binding buffer, 1 μl fluorescein-labelled annexin V and 1 μl propidium iodide were added. Then the cells were incubated for 5 min at room temperature (dark light). Finally, the cells were plated on glass slides and analysed by fluorescence microscopy (550 nm) for a total of 500 cells per sample, which allows detection even of a single apoptotic cell. To confirm the reduced DNA content (oligonucleosomal-size fragments) the cells stimulated with hormones, without and with staurosporine, after fixation were stained with intercalating dyes (propidium iodide) and were analysed by flow analysis cytometry scatter (Becton-Dickinson-Immunocytometry Systems, Erembodegem, Belgium).

### Immunocytochemistry

THP-1 cells were harvested at a concentration of 5 × 10^3^, being sedimented on poly-L-lysine-coated glass slides for 40 min at 4°C. The spots were then air-dried and fixed in cold acetone for 30 s, and stored at -20°C until the immunodetermination of poly-ADP ribose polymerase (PARP)-cleaved expression, proliferating cell nuclear antigen (PCNA) expression, NF-κB, IκB-α and IκB-α-ser 32 (Santa Cruz Biotechnology, Santa Cruz, CA, USA). After rehydration in PBS, spots were incubated with the anti-human antibody at different dilutions at room temperature. The second and third steps were performed using the improved biotin-streptavidin-amplified detection system (Vector Laboratories Inc., Burlingame, CA, USA). Briefly, according to this method, cells were incubated with the secondary antibody (biotinylated IgG, 1:20 dilution) for 20 min at room temperature and then, after several washes in PBS, cells were treated with the concentrated enzyme label (biotin-streptavidin-peroxidase) for 20 min and, finally, incubated at room temperature with the peroxidase-substrate solution (0.04% 3,3-diaminobenzidine [Sigma-Aldrich] in 50 mM Tris-HCl buffer containing 0.3% hydrogen peroxide) for 15 min. After rinsing with PBS, slides were counterstained with haematoxylin, were dried and cover slipped with Eukitt, and were examined by light microscopy and computerized image analysis. Controls were treated identically, except for omitting the primary or secondary antibodies.

### Image analysis

Image analysis was performed with the Leica Q500 MC Image Analysis System (Leica, Cambridge, UK). For each sample, 100 cells were randomly analysed and the pixels per micron square (positive area) were quantified by the Leica Q500 software. The single cells were randomly selected by the operators using the cursor and then automatically measured as the positive area. A constant optical threshold and filter combination was set to select only the positive cells.

### Statistical analysis

The results were analysed by the analysis of variance non-parametric test (Bonferroni test), and the values are presented as means ± standard deviations.

### Western blot analysis

Cells were lysed in buffer containing 20 mM Tris-HCl, 150 mM NaCl, 1 mM phenylmethylsulfonylfluoride, 5 mg/ml aprotinin, 0.5% Nonidet P-40 (Sigma-Aldrich) for 1 hour at 4°C. The lysates were centrifuged for 10 min at 13,000 rpm. The surnatants were collected and stored at -80°C.

The samples of surnatants were thereafter diluted with reducing sample buffer and were separated by electrophoresis on a 10% SDS-PAGE gel (20 μg protein per lane loaded). The proteins were transferred onto Hybond-C-nitrocellulose membrane (Amersham Italia, Milan, Italy). The reaction was blocked by DPBS with 5% non-fat powdered milk at 4°C overnight. For immunoblot analysis, the membranes were incubated with the different antibodies for 1 hour (NF-κB, IκB-α, IκB-α-ser 32, respectively; Santa Cruz Biotechnology) (dilution 1:200) in DPBS at room temperature with constant shaking, and were washed extensively in 0.05% DPBS/Tween 20, pH 7.4. Finally, the membranes were incubated with secondary horseradish peroxidase-labelled polyclonal anti-goat IgG antibody (SC-2020; Santa Cruz Biotechnology) (dilution 1:5000) in DPBS for 1 hour at room temperature. After washing three times in DPBS, the bands were detected using the enhanced chemiluminescence system (Amersham Italia).

### DNA binding assay (electrophoretic mobility shift assay)

Double-stranded oligonucleotides corresponding to the wild-type and mutated NF-κB consensus element (Santa Cruz Biotechnology) were used as ^32^P-labelled probes or as unlabelled competitors (100 times). The assays were performed in a final volume of 20 μl containing 10 mM Tris-HCl (pH 7.5), 50 mM NaCl, 1 mM dithiothreitol, 1 mM ethylenediamine tetraacetic acid and 10% glycerol. Briefly, 7 μg cell nuclear extracts, unstimulated or stimulated with testosterone and E2 as previously described, were pre-incubated with 4 μg poly(dI-dC) (Pharmacia, Milan, Italy) as a non-specific competitor, for 10 min at room temperature. End-labelled oligonucleotides (10 fmol, about 30,000 cpm) were then added to each mixture to a final volume of 20 μl and were incubated for an additional 20 min. The bound/retarded complexes were separated from the free probe by electrophoresis on 5% polyacrylamide gels and were visualized by autoradiography of the dried gels. A lane without nuclear extract was also included as the negative control. Competition experiments were performed in the presence of a 100-fold molar excess of unlabelled wall-type or mutated NF-κB oligonucleotide, added to the pre-incubation step.

For the super shift assay, 2 μl anti-p65, c-rel and p50 NF-κB subunit antibodies (Santa Cruz Biotechnology) were included in the pre-incubation mixture prior to the addition of the probe. A lane containing pre-immune serum was always included as the control.

## Results

### Effect of hormonal treatment on cell growth

**Figure 1:**
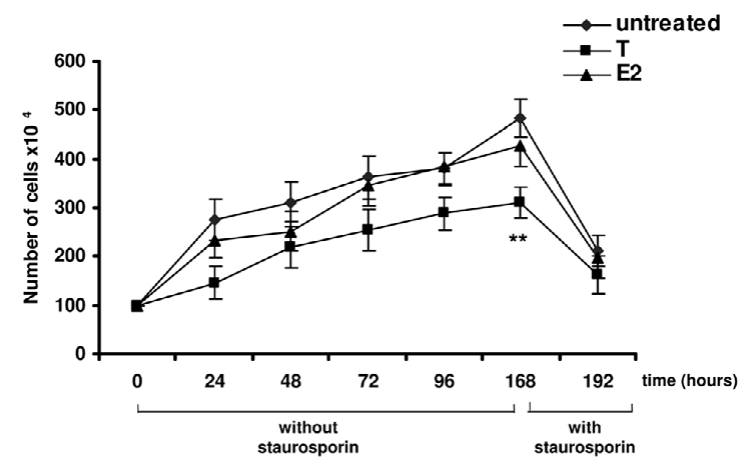
Growth rate of the cultured human THP-1 cell line without or with 17β-oestradiol (E2) and testosterone (T), and with staurosporine after 168 hours. The number of recovered live cells at different times was evaluated using the methyl tetrazolium salt reduction test. Results are expressed as the mean ± standard deviation of five different experiments.

As already stated, hormonal signals regulate the amount of the cell-cycle control proteins and of the transcription factors [26]. In the present study, after 168 hours we observed a significant growth inhibition of the human monocytic cell line activated with IFN-γ and treated with testosterone, when compared with the other conditions studied. At 168 hours the cell growth was reduced by 14.8% versus untreated controls and by 12.7% versus E2-treated cells. These data were obtained with the methyl-tetrazolium salt test and confirmed with the trypan blue exclusion assay (Fig. 1). The bromodeoxyuridine/propidium iodide incorporation test showed similar results (data not shown).

**Figure 2:**
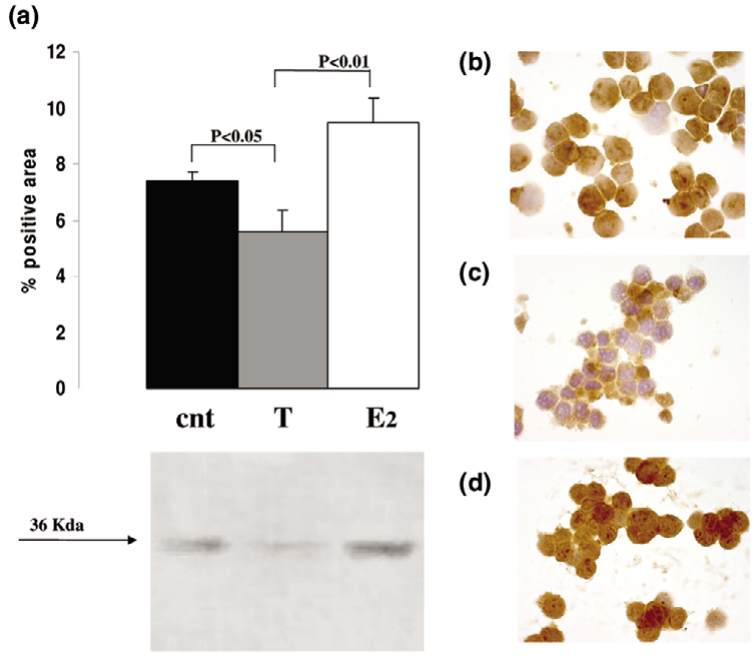
Proliferating cell nuclear antigen (PCNA) expression in cultured human THP-1 cell line. **(a) **Proliferating cell nuclear antigen (PCNA) expression in the cultured human THP-1 cell line after hormonal treatment. Results are expressed as the mean ± standard deviation of the percentage of staining area (positive area defined as the number of pixels detectable per micron square) for 100 cells in five different experiments. Bottom: western blot analysis. **(b) **PCNA expression in untreated THP-1 cells (control [cnt]). **(c) **PCNA expression in THP-1 cells treated with testosterone (T) after 168 hours. **(d) **PCNA expression in THP-1 cells treated with 17β-oestradiol (E2) after 168 hours (magnification, × 500).

Further interesting data were related to the modulation of the PCNA expression, as a marker for proliferating cells. This marker was analysed by immunocytochemistry and the data were confirmed by western blot analysis. In the testosterone-treated cells, the expression of the PCNA at the immunocytochemistry analysis was found decreased by 24% when compared with untreated cells, and was decreased by 40% when compared with E2-treated cells (Fig. 2a–d). Interestingly, the testosterone-treated cells showed staining positivity localized in the cell cytoplasm, whereas in the untreated and E2-treated cells the PCNA expression was found predominantly in the nucleus.

### Effects of hormonal treatment on cell apoptosis

In the present study the inhibitory role of E2 on apoptosis, as well as the pro-apoptotic effects exerted by testosterone in long-term treatment of THP-1 cells, were investigated.

**Figure 3:**
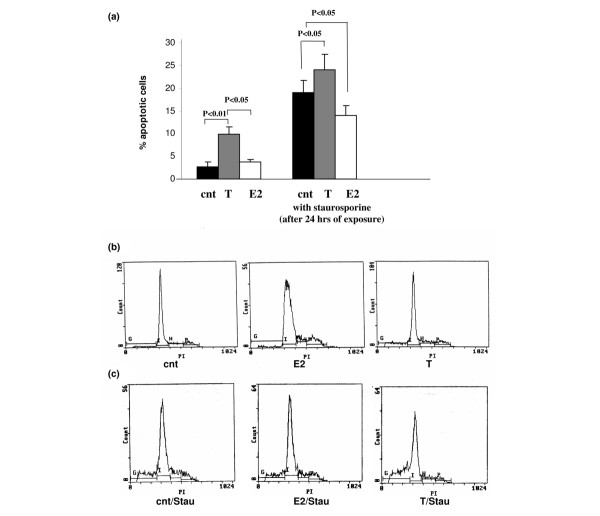
Apoptosis evaluation in cultured human THP-1 cell line. **(a) **Apoptosis evaluation in the cultured human THP-1 cell line after 168 hours of hormonal treatment without and with staurosporine. Results are expressed as the mean ± standard deviation of the percentage number of annexin-positive cells in five different experiments. **(b) **Apoptotic DNA evaluation in THP-1 cells after 168 hours of hormonal treatment without staurosporine as detected by flow analysis cytometry scatter analysis. Region G, control (cnt), 3.96%; 17β-oestradiol (E2), 5.54%; testosterone (T), 15.9%. **(c) **Apoptotic DNA evaluation in THP-1 cells after 168 hours of hormonal treatment and staurosporine (stau) for 24 hours, as detected by flow analysis cytometry scatter analysis. Region G: control/S, 18.9%; E_2_/S, 16.5%; T/S, 24.5%. PI, propidium iodide.

The annexin V-propidium iodide analysis showed a significant increase of early and late apoptosis in testosterone-treated cells when compared with other conditions. In the absence of staurosporine, the apoptosis was found increased in testosterone-treated cells (9.9%) when compared with control untreated cells (2.7%) and with E2-treated cells (3.8%). In the presence of staurosporine, the apoptotic cells were increased in all conditions. In testosterone-treated cells (24.8%) the apoptosis was found increased when compared with untreated cells (19.4%). On the contrary, in the E2-treated (15%) cells the apoptosis was decreased versus controls (19.4%) (Fig. 3a). These data were confirmed by flow analysis cytometry scatter analysis (Fig. 3b,c).

**Figure 4:**
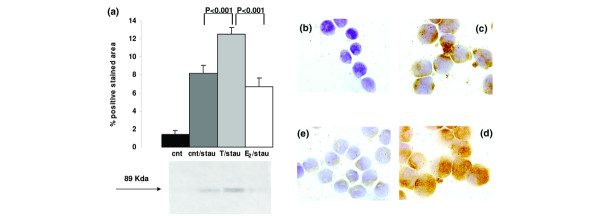
Poly-ADP-ribose polymerase (PARP)-cleaved expression in cultured human THP-1 cell line. **(a) **Poly-ADP-ribose polymerase (PARP)-cleaved expression in the cultured human THP-1 cell line in basal conditions and after 168 hours of hormonal treatment and staurosporine (stau). Results are expressed as the mean ± standard deviation of the percentage of the positive area for 100 cells in five different experiments. Bottom: western blot analysis related to PARP-cleaved expression in untreated cells (basal condition) and cells treated with hormones and staurosporine. **(b) **PARP-cleaved expression in THP-1 cells (basal condition). **(c) **PARP-cleaved expression in THP-1 cells after normal medium. **(d) **PARP-cleaved expression in THP-1 cells after testosterone (T) treatment. **(e) **PARP-cleaved expression in THP-1 cells after 17β-oestradiol (E2) treatment. cnt, control.

The immunocytochemistry analysis showed a significant increase of the PARP-cleaved form in testosterone-treated cells compared with other conditions, suggesting an increase of pro-apoptotic events. On the contrary, the PARP-cleaved staining showed a decrease (19% versus untreated controls) in E2-treated cells, suggesting a trend of oestrogens in protecting cells from the apoptotic stimuli (Fig. 4a–e). These data were confirmed with western blot analysis.

### Effects of hormonal treatment on the NF-κB complex

**Figure 5:**
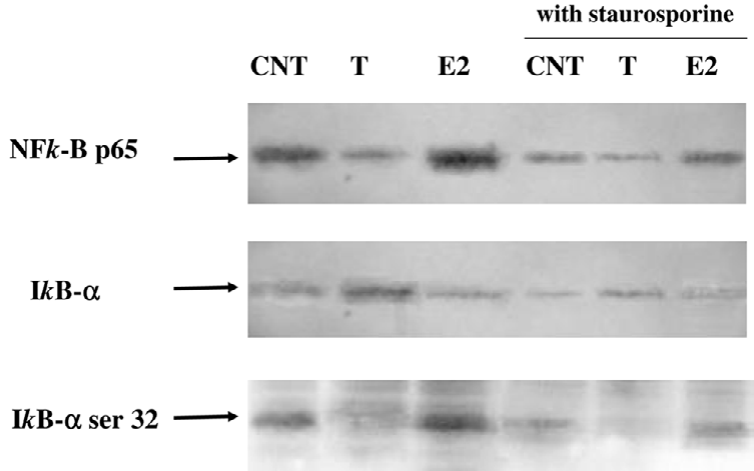
NF-κB p65, IκB-α and phosphorylated IκB-α (ser 32) protein expression in the cultured human THP-1 cell line after 168 hours of hormonal treatment without or with staurosporine. The results are representative of four separate experiments. CNT, control; T, testosterone; E2, 17β-oestradiol.

**Figure 6:**
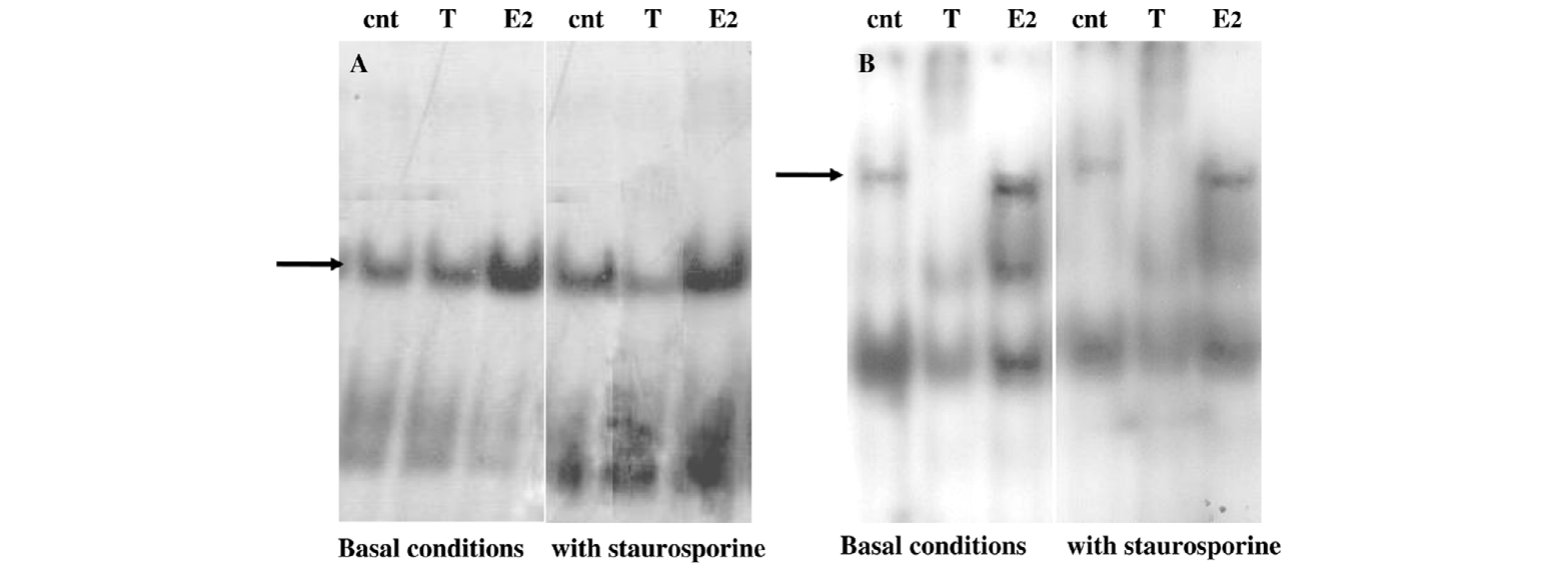
Electrophoretic mobility shift assay for NF-κB (arrow) in the cultured human THP-1 cell line. **(a) **Electrophoretic mobility shift assay for NF-κB (arrow) in the cultured human THP-1 cell line after 168 hours of hormonal treatment and without or with staurosporine (17 nM) reveals an increased DNA binding in 17β-oestradiol (E2)-treated cells. **(b) **Super shift assay for p65 (arrow) in the cells under the same conditions confirms the increased binding of p65 in E2-treated cells. The results are representative of four separate experiments. cnt, control; T, testosterone.

The defined mechanism of NF-κB activation includes the site-specific phosphorylation and subsequent degradation of the inhibitory IκB-α factor, which usually retains the NF-κB factors inactivated in cytosol [27]. We therefore performed western blot analysis to detect the expression of NF-κB (p65), IκB-α and the IκB-α phosphorylated form (IκB-α-ser 32) in THP-1 cells treated with the different hormone combinations. As shown in western blot analysis (Fig. 5) a decreased expression of NF-κB (p65) and an increased content of IκB-α with a concomitant decrease of the IκB-α phosphorylated form was observed in testosterone-treated cells when compared with untreated controls and with E2-treated THP-1 cells; the differences between conditions observed with staurosporine treatment were similar but the expression was lower. The observed differences between E2-treated and testosterone-treated THP-1 cells were confirmed by immunocytochemistry staining and image analysis; similar data were observed with staurosporine treatment (Table 1). The concomitant electrophoretic mobility shift assay analysis for the evaluation of the NF-κB DNA binding confirmed an increased binding in E2-treated cells, when compared with other conditions with or without staurosporine, as shown in Fig. 6a. Finally, the gel shift assay of nuclear protein extracts from E2-treated cells again showed an increase of the p65 subunit DNA binding (Fig. 6b) when compared with other conditions. Similar results were obtained during staurosporine treatment.

## Discussion

Monocytes/macrophages contribute to the autoimmune process, mainly acting as antigen-processing/presenting cells and sources of inflammatory cytokines, particularly at the level of the synovial tissue in RA [28]. Moreover, sex hormones can exert local actions (paracrine) in the tissues in which they are formed, including the synovial tissue [29,30].

Activated THP-1 cells differentiate into macrophages for long-term cultures. On the contrary, synovial macrophages are characterized by a short life during *in vitro *culture.

The present study shows opposite effects by sex hormones on cultures of activated monocytic/macrophage cells (THP-1 cells) concerning their modulatory effects on cell proliferation and/or apoptosis.

The signalling pathways modulating the pro-inflammatory and anti-inflammatory mechanisms seem to involve steroidal hormone receptor activation and the NF-κB complex factors, In addition, oestrogens may differently regulate NF-κB activation depending on the cell type tested [14,31].

E2 therefore increased the expression of markers of cell growth and proliferation, whereas testosterone induced an increase of the PARP-cleaved expression, indicating DNA damage and apoptosis. In addition, to support the proliferative role exerted by E2, the THP-1 cells pre-treated with the oestrogens showed a decrease of staurosporine-induced apoptosis when compared with testosterone-treated and untreated cells. Furthermore, the increased NF-κB p65 expression and the evident NF-κB binding to DNA in E2-treated cells, when compared with untreated cells or with testosterone-treated cells, as well as the increased levels of the IκB-α phosphorylated form, seems to support the major enhancing role exerted by oestrogens on the immune/inflammatory response by activating the NF-κB complex.

On the contrary, the observed positive upregulation of IκB-α exerted by testosterone treatment presumably dampens the pro-inflammatory effects mediated by the NF-κB activation, and therefore might represent a further mechanism by which androgens exert anti-inflammatory effects. Recent studies support these results, showing that E2 inhibits apoptosis in different cell types (cardiac myocytes and others) whereas androgens have been found to induce apoptosis [32,33].

The increased concentrations of oestrogens (and low androgens) recently described at the level of the synovial fluid of RA patients of both sexes [29] seem to support their possible modulator roles on synovial tissue hyperplasia and chronic synovial cell activation, by considering the oestrogenic effects on cell proliferation and apoptosis. These observations have been recently obtained also in human breast cancer cells [34].

To explain increased oestrogen concentrations in RA synovial fluids, the pro-inflammatory cytokines (tumour necrosis factor alpha, IL-1β, IL-6) have been found to accelerate the metabolic conversion of oestrogens from androgens by inducing the synovial tissue aromatases [35-37]. As a consequence, locally increased oestrogen levels might exert activating effects on synovial cell proliferation, including macrophages and fibroblasts [38].

## Conclusion

In the present study the concentrations for E2 and testosterone tested seem to modulate the activity of NF-κB molecules in the human monocytic/macrophage cell line (THP-1) with opposite effects, interfering with cell growth and apoptosis. These observations might provide a further biological link between gender effects and the complex inflammatory process involved in rheumatoid synovitis. Further studies, using peripheral metabolites of oestrogens and synovial macrophages from RA patients, might extend the value of these observations.

## Abbreviations

DPBS = Dulbecco's phosphate-buffered saline; E2 = 17β-oestradiol; IFN = interferon; IL = interleukin; NF = nuclear factor; PARP = poly-ADP ribose polymerase; PBS = phosphate-buffered saline; PCNA = proliferating cell nuclear antigen; RA = rheumatoid arthritis; THP-1 = cultured human monocytic/macrophage cell line.

## Competing interests

The author(s) declare that they have no competing interests.

## Authors' contributions

MC conceived the study and drafted the manuscript. SC participated in conducting THP-1 cell growth and the functional assay. PM conducted the immunocytochemistry assay and the western blot assay. PG conducted the electrophoretic mobility shift assay and the super shift assay. AS helped to perform statistical analysis. BV participated in the study design, coordination and data analysis, and drafted the manuscript. All authors read and approved the final manuscript.

## Figures and Tables

**Table 1:** NF-κB complex, IκB-α and IκB-α-ser 32 evaluated by immunocytochemistry

	NF-κB	IκB-α	IκB-α-ser 32
Without staurosporine			
Control	42.6 ± 4	17.7 ± 8	17.1 ± 2
Testosterone (10 nM)	29.6 ± 5 (T vs control, *P *< 0.001)	26.9 ± 4 (T vs control, *P *< 0.01)	13 ± 3
17β-oestradiol (10 nM)	45.8 ± 3 (E2 vs T, *P *< 0.001)	12.5 ± 2 (E2 vs T, *P *< 0.001)	25.3 ± 2 (E2 vs T, *P *< 0.01; E2 vs control, *P *< 0.001)
With staurosporine			
Control	18.9 ± 4	23 ± 6	11.6 ± 4
Testosterone (10 nM)	18.2 ± 4	20.7 ± 3	9.2 ± 2
17β-oestradiol (10 nM)	32.3 ± 6 (E2 vs T, *P *< 0.05; E2 vs control, *P *< 0.001)	25.5 ± 4 (E2 vs T, *P *< 0.05)	22.8 ± 3 (E2 vs T, *P *< 0.001; E2 vs control, *P *< 0.001)

Expression and regulation after 168 hours of hormonal treatment (T, testosterone; E2, 17β-estradiol) in THP-1 cells (without and with staurosporine). Data are expressed as the mean ± standard deviation of the positive area per 100 cells stained for NF-κB complex, IκB-α and IκB-α-ser 32. Results were obtained as the mean of five different experiments. The statistical evaluation was determined for T versus control, for E2 versus control, and for E2 versus T (absence of significance not reported).
